# Tuberculosis and diabetes mellitus comorbidity in an adult Ugandan population

**DOI:** 10.1186/s12879-024-09111-8

**Published:** 2024-02-22

**Authors:** Davis Kibirige, Irene Andia-Biraro, Ronald Olum, Susan Adakun, Stella Zawedde-Muyanja, Christine Sekaggya-Wiltshire, Ivan Kimuli

**Affiliations:** 1 Department of Medicine, Uganda Martyrs Hospital Lubaga, Kampala, Uganda; 2 Non-communicable Diseases Program, Medical Research Council/Uganda Virus Research Institute and London School of Hygiene and Tropical Medicine Uganda Research Unit, Entebbe, Uganda; 3 Department of Medicine, Makerere University College of Health Sciences, Kampala, Uganda; 4 Makerere University School of Public Health, Kampala, Uganda; 5 Adult Tuberculosis ward, Mulago National Referral and Teaching Hospital, Kampala, Uganda; 6 The Infectious Diseases Institute, College of Health Sciences, Makerere University Kampala, Kampala, Uganda; 7 Department of Physiology, College of Health Sciences, Makerere University Kampala, Kampala, Uganda

**Keywords:** Tuberculosis, Diabetes mellitus, Characteristics, Associated factors, Uganda, Sub-saharan Africa

## Abstract

**Background:**

Diabetes mellitus (DM) has a direct impact on the clinical manifestation and prognosis of active tuberculosis disease (TB) and is known to increase the chance of developing the condition. We sought to determine the prevalence of DM in adult Ugandan patients with recently diagnosed TB and the associated sociodemographic, anthropometric, and metabolic characteristics of TB-DM comorbidity.

**Methods:**

In this cross-sectional study conducted at the adult TB treatment centres of three tertiary healthcare facilities in Uganda, we screened adult participants with recently diagnosed TB (diagnosed in < 2 months) for DM. All participants were screened with five tests; initially with a random blood glucose (RBG) test, and then later with fasting blood glucose (FBG), laboratory-based glycated hemoglobin (HbA1c), point-of-care (POC) HbA1c, and oral glucose tolerance test (OGTT) if the RBG was ≥ 6.1 mmol/l. The WHO guidelines for diagnosing and managing DM were used to support the DM diagnosis. To identify the factors associated with DM-TB comorbidity, logistic regression was used.

**Results:**

A total of 232 participants with recently diagnosed TB were screened for DM. Of these, 160 (69%) were female. The median (IQR) age, body mass index, and RBG of all study participants was 35 (27–42) years, 19.2 (17.6–21.3) kg/m2, and 6.1 (5.5–7.2) mmol/l, respectively. About half of the participants (*n* = 117, 50.4%) had RBG level ≥ 6.1 mmol/l. Of these, 75 (64.1%) participants returned for re-testing. Diabetes mellitus was diagnosed in 32 participants, corresponding to a prevalence of 13.8% (95% CI 9.9–18.9). A new diagnosis of DM was noted in 29 (90.6%) participants. On logistic regression, age ≥ 40 years was associated with increased odds of TB and DM comorbidity (AOR 3.12, 95% CI 1.35–7.23, *p* = 0.008) while HIV coinfection was protective (AOR 0.27, 95% CI 0.10–0.74, *p* = 0.01).

**Conclusion:**

TB and DM comorbidity was relatively common in this study population. Routine screening for DM in adult Ugandan patients with recently diagnosed TB especially among those aged ≥ 40 years and HIV-negative patients should be encouraged in clinical practice.

**Supplementary Information:**

The online version contains supplementary material available at 10.1186/s12879-024-09111-8.

## Introduction

Currently, Africa continues to experience a growing dual burden of diabetes mellitus (DM) and tuberculosis (TB), posing significant public health challenges [1, 2]. Compelling evidence shows that DM increases the odds of developing TB [3–5]. In the seminal systematic review and meta-analysis of 13 observational studies with > 1.7 million participants, DM was associated with a three-fold increase in the risk of developing TB [3]. Another systematic review and meta-analysis of 16 observational studies reported a pooled prevalence of DM of 9% in sub-Saharan African patients with TB [6].

Diabetes mellitus directly affects the clinical presentation and treatment outcomes of TB. Patients with TB and DM comorbidity often present with more lung cavitation on chest X-ray, smear-positive or culture-positive pulmonary TB, higher bacillary loads, and increased risk of TB recurrence, multi-drug resistant TB, and mortality [7–10].

Several sociodemographic, anthropometric, and clinical factors have been identified as determinants of DM in patients with TB. In one global systematic review and meta-analysis that evaluated the prevalence of DM in 2.3 million adult patients with TB, increasing age, male sex, and low TB burden were reported to be associated with increasing prevalence of DM [5]. Other cross-sectional studies have reported an association between TB-DM comorbidity and marital status, rural residence, family history of DM, female sex, overweight/obesity, and HIV coinfection [11–19].

Because of the close interaction between DM and TB, the International Union against TB and Lung Disease (The Union) advocates for proactive bidirectional screening and integrated management of DM and TB to improve early diagnosis, optimal management, and treatment outcomes [20, 21]. Following this recommendation, several studies have been performed globally on bidirectional screening and management of DM and TB.

In Uganda, studies that have investigated the prevalence of DM in adult patients with active TB disease have reported a prevalence ranging from 1.3 to 8.5% [22–27]. The heterogeneity in the study findings could be explained by the differences in patients studied (hospitalised vs. outpatient patients) and the diagnostic tests used (fasting blood glucose or FBG, glycated haemoglobin or HbA1c, and random blood glucose or RBG). None of these studies has screened for DM using more than two diagnostic blood tests (including the oral glucose tolerance test or OGTT which is the gold standard diagnostic test for DM), hence the definite burden of DM in patients with TB in Uganda may not be known.

To address this clinical gap, the tuberculosis and diabetes comorbidity (TADIC) study aimed to determine the true prevalence of DM in patients with recently diagnosed TB using five screening tests (RBG, FBG, laboratory-based HbA1c, point-of-care or POC HbA1c, and an OGTT). In addition, we also sought to describe the sociodemographic, clinical, anthropometric, and metabolic characteristics of patients with TB and DM comorbidity and to identify the factors associated with TB-DM comorbidity to guide targeted screening in clinical practice.

## Methods

### Study setting, duration, and participants

This study was performed in adult outpatient TB treatment centres of three tertiary healthcare facilities located in Central Uganda from January 2022 to January 2023. These three study sites were selected because they have highly functional TB treatment units that provide holistic TB care to large numbers of patients.

We recruited patients aged ≥ 18 years with a recent bacteriological, radiological, or clinical diagnosis of TB (< 2 months from the time of diagnosis), either treatment naïve or initiated on TB treatment, and were willing to provide written informed consent. We excluded patients who could not comply with the study visits. A total of 232 participants were enrolled in the study. The response rate of the participants at the screening stage was 91%.

### Assessment of socio-demographic, clinical, anthropometric, and metabolic characteristics

Following written informed consent, a pre-tested case report form was used by the study nurses to obtain relevant demographic (age, sex, residence, employment, marital status, level of education, family history of DM), social (smoking and alcohol use habits), and medical history (self-reported history of comorbidities like HIV and DM and glucose-lowering therapies being used, TB symptoms at the time of diagnosis, history of TB). A participant was classified as a former smoker if he or she used to smoke and later quit. Those classified as current smokers were participants who were still smoking at the time of recruitment. The medical records of the participants were also reviewed to document information on the category of TB diagnosed, the presence of resistance to rifampicin, and chest X-ray findings. The interpretation of the chest radiographs was performed by either a radiologist or radiographer.

Biophysical measurements which included resting blood pressure and relevant anthropometric measurements (weight, height, waist circumference [WC], hip circumference [HC], body mass index [BMI], and waist: hip circumference ratio [WHR]) were then performed while observing the privacy of the participants. Following the World Health Organisation (WHO) STEPwise guidelines of performing anthropometric measurements, the participants’ weight, height, WC, and HC were measured using a Seca© weighing scale, stadiometer, and tape measures respectively. The participants were advised to remove any heavy clothing, footwear, and headgear before performing these measurements. The waist circumference was measured at the approximate midpoint between the lower margin of the last palpable rib and the top of the iliac crest while the hip circumference was measured at the widest portion of the buttocks. The BMI was calculated using the formula: participant’s weight in kilograms divided by the square of the height in metres (kg/m^2^) and categorised based on the same guidelines [28].

### Assessment of the glycaemic status

**Fig. 1:**
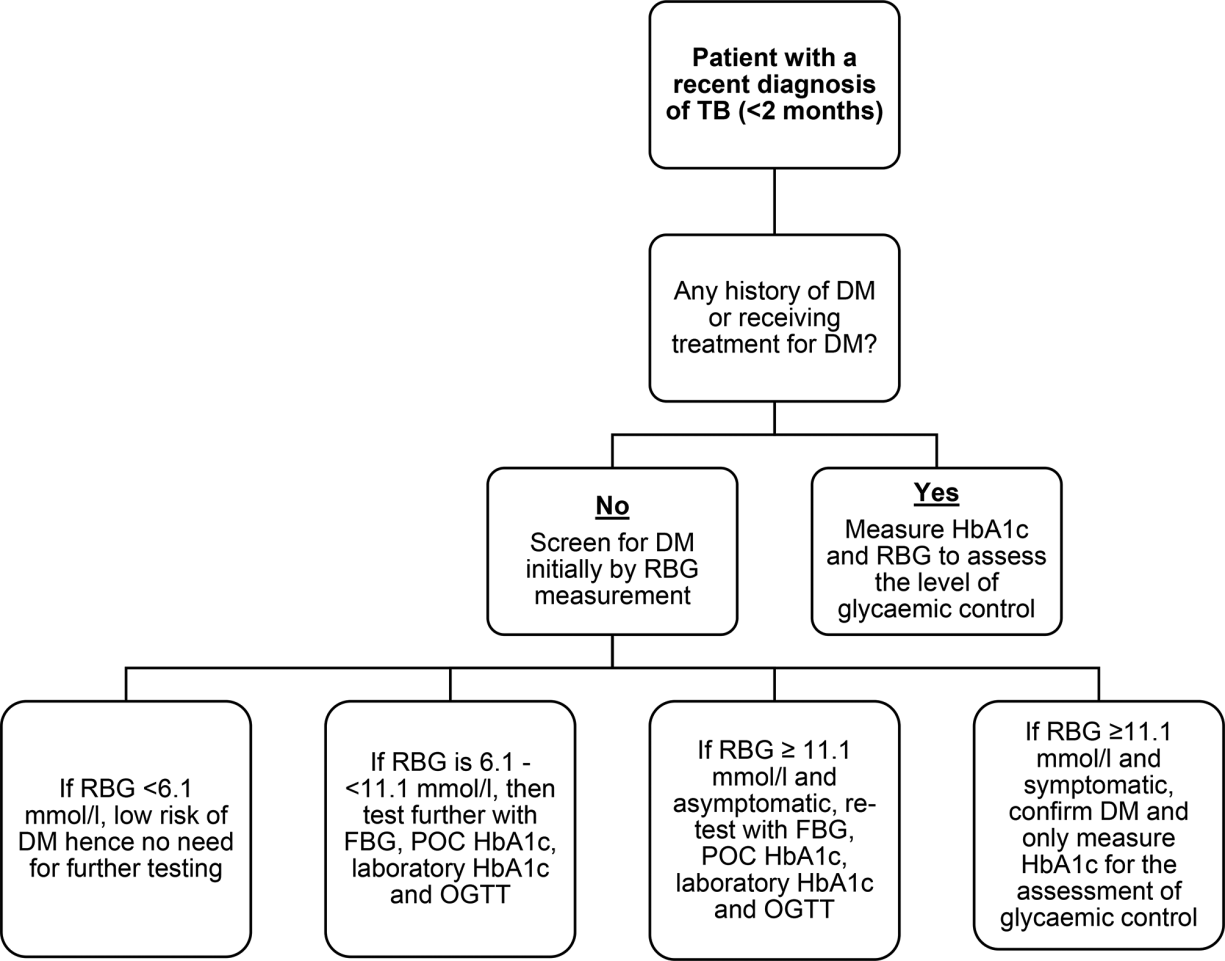
Flowchart summarising the study screening pathway

Figure 1 summarises the study screening process.

The Union and WHO guidelines were used to guide the screening and diagnosis of DM [20, 29]. The initial screening test for all study participants regardless of DM status involved measuring the RBG level with a One Touch Select Plus Flex® glucometer (TA-2019/733, Lifescan IP Holdings, LLC, USA). For participants with a self-reported history of DM, HbA1c measurement was performed to assess long-term glycaemic control status. No further assessment of the glycaemic status was undertaken.

Participants without a prior self-reported history of DM and RBG of < 6.1 mmol/l were deemed to have a low risk of developing diabetes and did not also undergo further blood glucose testing. The participants whose RBG level was ≥ 6.1 mmol/l and < 11.1 mmol/l had FBG, laboratory-based and POC HbA1c measurement, and a 75-gram OGTT performed within 2 weeks to confirm the status of DM. Participants who had an RBG level ≥ 11.1 mmol/l and symptoms like polyuria and polydipsia, which are strongly suggestive of DM, were diagnosed with DM and underwent HbA1c testing as an additional test to determine their level of glycaemic control. All participants that had RBG level ≥ 11.1 mmol/l and were asymptomatic were scheduled for FBG, laboratory-based and POC HbA1c measurement, and a 75-gram OGTT to confirm their glycaemic state. For the participants who underwent an OGTT, only the fasting and 2-hour blood glucose levels were measured.

The laboratory HbA1c measurement was done using an electro-chemiluminescence immunoassay manufactured by Roche Diagnostics Limited, Germany on a Cobas 6000 C-model SN 14H3-15 machine (Hitachi High Technologies Corporation, Tokyo Japan) while the POC HbA1c measurement was performed using quantitative immune-chromatography on an SD Biosensor A1c Care™ HbA1c analyser (SD Biosensor Inc, Seoul, Republic of South Korea).

### Additional metabolic tests performed

All participants who returned for re-testing had a fasting blood sample drawn for measurement of alanine transaminase, fasting insulin, and C-peptide concentrations and estimate the HOMA2 insulin resistance (HOMA2-IR) and HOMA2 beta cell function (HOMA2-%B) using the Diabetes Trial Unit’s online HOMA calculator [30]. The HOMA2-IR and HOMA2-%B, in addition to the fasting C-peptide concentration, were used as surrogate markers of insulin resistance and pancreatic beta-cell function, respectively. All the above laboratory tests were performed at the South African National Accreditation System (SANAS)-accredited Clinical Chemistry laboratory at the Uganda Martyrs Hospital Lubaga, Kampala Uganda.

### Definition of study outcomes

Diabetes mellitus was defined as either one of the following according to the WHO guidelines for diagnosing DM: (1) pre-existing history of DM (either treatment naïve or on any glucose-lowering therapy); (2) FBG ≥ 7 mmol/l; (3) a 2-hour blood glucose level after a 75-gram OGTT of ≥ 11.1 mmol/l; (4) laboratory-based or POC HbA1c level ≥ 6.5% or 48 mmol/mol. Prediabetes was defined as either (1) FBG level of 5.6–6.9 mmol/l; (2) a 2-hour blood glucose level after a 75-gram OGTT of 7.8–11.1 mmol/l; (3) laboratory-based or POC HbA1c level of 5.7–6.4% (39–47 mmol/mol). Study participants with normoglycaemia were those with RBG < 6.1 mmol/l on initial screening or FBG < 5.6 mmol/l, laboratory-based or POC HbA1c < 5.7% (mmol/mol), and a 2-hour blood glucose level after a 75-gram OGTT of < 7.8 mmol/l on re-testing [29].

### Sample size estimation

The sample size was calculated based on Leslie Kish’s formula for cross-sectional studies (1965) [31]: N = P (1-P) Z^2^ / d^2^, where P is the prevalence of DM among adult Ugandan patients with TB receiving outpatient treatment of 2.3% [26], Z = 1.96 (the Z score corresponding to 95% confidence interval), and d the margin of error of 2%. This gave a sample size of 215 which was increased to 232 participants to cater for loss to follow-ups.

### Statistical analysis

To describe the characteristics of all study participants, we used proportions for the categorical variables and medians with inter-quartile range (IQR) for the continuous variables. The categorical and continuous variables were compared using chi-square (or Fisher’s exact test where appropriate) and Kruskal Wallis tests, respectively. Proportions and their corresponding 95% confidence intervals (CI) were used to describe the participants with confirmed DM, prediabetes, and normoglycaemia. The differences in the socio-demographic, clinical, anthropometric, and metabolic characteristics of participants with and without TB and DM comorbidity were analysed using the chi-square test for categorical data and the Kruskal Wallis test for continuous data because the latter was not normally distributed.

Specific variables informed by the univariate logistic regression model with a p-value of 0.2 and also reported in the literature to be associated with TB and DM comorbidity were added to the final logistic regression model to identify the factors associated with TB and DM comorbidity in this study population. All analyses were done using STATA statistical software version 15 College Station, TX: StataCorp LLC.

## Results

### Baseline characteristics of all study participants

Table 1 summarises the sociodemographic, clinical, anthropometric, and metabolic characteristics of all participants and those with and without TB and DM comorbidity. The median (IQR) age, body mass index, and RBG of all study participants was 35 (27–42) years, 19.2 (17.6–21.3) kg/m^2^, and 6.1 (5.5–7.2) mmol/l, respectively, with 69% of participants being females. HIV co-infection, smear-positive, and smear-negative pulmonary TB were reported in 90 (38.8%) participants, 193 (87.3%) participants, and 17 (7.7%) participants, respectively. Resistance to rifampicin on GeneXpert testing was documented in 36 (18.5%) participants.

**Table 1:** Sociodemographic, clinical, anthropometric and metabolic characteristics of all participants, and those with and without tuberculosis and diabetes comorbidity

Characteristic	All participants (*n* = 232)	TB with DM (*n* = 32, 13.8%)	TB without DM (*n* = 200, 86.2%)	P-value
**Age (years)**	35.0 (27.0–42.0)	42.5 (37.0–53.5)	33.5 (25.0–42.0)	< 0.001
**Sex**				
Males	72.0 (31.0)	6.0 (18.8)	66.0 (33.0)	0.12
Females	160.0 (69.0)	26.0 (81.2)	134.0 (67.0)	
**Residence**				
Urban	181 (79.4)	22 (68.8)	159 (79.1)	0.04
Semi-urban	35 (15.4)	9 (28.1)	26 (12.9)	
Rural	12 (5.3)	0 (0)	12 (6)	
**Employment**				
Employed (skilled/professional)	20 (8.7)	5 (15.6)	15 (7.5)	0.07
Employed (unskilled)	139 (60.4)	23 (71.9)	116 (57.7)	
Unemployed	63 (27.4)	4 (12.5)	59 (29.4)	
Student	8 (3.5)	0 (0)	8 (4)	
**Level of education**				
Illiterate	17 (7.4)	4 (12.5)	13 (6.5)	0.27
Primary	85 (37)	12 (37.5)	73 (36.3)	
Secondary	101 (43.9)	15 (46.9)	86 (42.8)	
Tertiary	27 (11.7)	1 (3.1)	26 (12.9)	
**Marital status**				
Single	122 (53)	14 (43.8)	108 (53.7)	0.02
Married	82 (35.7)	14 (43.8)	68 (33.8)	
Divorced	17 (7.4)	0 (0)	17 (8.5)	
Widow/widower	9 (3.9)	4 (12.5)	5 (2.5)	
**Familial history of diabetes**				
Yes	22 (12.0)	3 (11.1)	19 (12.1)	0.88
No	162 (88.0)	24 (88.9)	138 (87.9)	
**Smoking history**				
Yes	12 (5.2)	0 (0)	12 (6)	0.04
No	175 (75.4)	21 (65.6)	154 (76.6)	
Quit	45 (19.4)	11 (34.4)	34 (16.9)	
**Alcohol ingestion history**				
Yes	46 (20.2)	8 (25)	38 (18.9)	0.06
No	111 (48.7)	9 (28.1)	102 (50.7)	
Quit	71 (31.1)	14 (43.8)	57 (28.4)	
**Comorbidities**				
HIV co-infection	90 (38.8)	6 (18.8)	84 (41.8)	0.01
**TB symptoms**				
Cough	219 (94.8)	31 (96.9)	188 (93.5)	0.57
Coughed blood	45 (19.6)	5 (15.6)	40 (19.9)	0.55
Fever	107 (46.3)	13 (40.6)	94 (46.8)	0.49
Night sweats	128 (55.2)	18 (56.3)	110 (54.7)	0.16
Weight loss	183 (78.9)	25 (78.1)	158 (78.6)	0.88
Loss of appetite	125 (53.9)	20 (62.5)	105 (52.2)	0.29
Lymph nodes	27 (11.6)	3 (9.4)	24 (11.9)	0.67
**Past history of TB**				
Yes	41 (18)	7 (21.9)	34 (16.9)	0.48
No	186 (81.6)	24 (75)	162 (80.6)	
**Category of TB**				
Smear-positive TB	193 (87.3)	23 (71.9)	170 (84.6)	0.10
Smear-negative TB	17 (7.7)	5 (15.6)	12 (6)	
Extra-pulmonary TB	11 (5)	2 (6.3)	9 (4.5)	
**Resistance to rifampicin**				
Yes	36 (18.5)	4 (12.5)	32 (15.9)	1.00
No	157 (80.5)	21 (65.6)	136 (67.7)	
**Findings on CXR**				
Solitary cavity	2 (1.5)	0 (0)	2 (1)	0.57
Multiple cavities	66 (48.9)	8 (25)	58 (28.9)	0.64
Lower lobe infiltrates	50 (37)	7 (21.9)	43 (21.4)	0.96
Pleural effusion	17 (12.6)	3 (9.4)	14 (7)	0.63
**Anthropometry**				
Weight (kg)	53.5 (48.0–60.0)	53.4 (48.0–62.4)	53.7 (48.0–60.0)	0.73
Height (cm)	166.0 (160.1–172.0)	165.1 (161.8–173.7)	166.3 (160.0–171.7)	0.47
BMI (kg/m2)	19.2 (17.6–21.3)	19.0 (17.5–22.5)	19.2 (17.8–21.3)	0.97
WC (cm)	75.0 (70.5–79.4)	77.0 (72.0–86.5)	75.0 (70.0–79.0)	0.11
HC (cm)	84.0 (79.0–90.0)	84.5 (80.0–94.0)	84.0 (79.0–90.0)	0.41
WHR	0.89 (0.83–0.94)	0.90 (0.86–0.96)	0.88 (0.93 − 0.82)	0.11
Systolic BP (mmHg)	116 (105–127)	113 (106–130)	116 (105–126)	0.84
Diastolic BP (mmHg)	77 (70–84)	77 (74–87)	77 (69–84)	0.20
**Metabolic**				
ALT (U/L)	16 (13–22)	16.5 (13–22)	16 (13–22)	0.63
RBG (mmol/l)	6.1 (5.5–7.2)	7.7 (6.9–9.4)	6.0 (5.4–6.8)	< 0.001
FBG (mmol/l)	6.3 (5.8–7.0)	7.3 (7.0–7.9)	6.0 (5.5–6.4)	< 0.001
2-hour BG (mmol/l)	7.8 (6.8–9.0)	8.8 (7.7–12.0)	7.0 (5.9–8.3)	< 0.001
Laboratory-based HbA1c (%)	5.4 (5.0–6.0)	5.8 (5.2–6.7)	5.3 (4.9–5.9)	0.005
POC HbA1c (%)	5.6 (5.2–6.1)	5.9 (5.6–6.2)	5.5 (5.0–5.9)	0.001

### Glycaemic profile of the participants

Of the 232 participants enrolled in the study and initially screened for DM using RBG measurement, 117 participants (50.4%) had RBG level ≥ 6.1 mmol/l and were scheduled to return for further evaluation. Of these, 75 (64.1%) participants returned for additional blood glucose testing.

A total of 135 (58.2%) participants, 65 (28%) participants, and 32 (13.8%) participants had diagnoses of normoglycemia, prediabetes, and DM, respectively. Of the participants re-tested, an abnormal RBG, POC HbA1c, 2-hour glucose, laboratory-based HbA1c, and FBG level was used to diagnose DM in five (2.2%) participants, three (4.1%) participants, seven (9.5%) participants, eight (10.7%) participants, and 22 (29.3%) participants, respectively. Only three (9.4%) of the 32 participants diagnosed with DM had a pre-existing history of DM and were on oral medication (metformin and a sulfonylurea), with all having a median HbA1c level ≥ 10% (86 mmol/mol) (summarised in Supplementary Tables 1 and Supplementary Fig. 1).

### Socio-demographic, clinical, and metabolic characteristics of participants with and without tuberculosis and diabetes mellitus comorbidity

Compared with those with TB alone, participants with TB and DM comorbidity were more likely to be older (median [IQR]: 42.5 [37.0–53.5] years vs. 33.5 [25.0–42.0] years, *p* < 0.001), to reside in a semi-urban area (28.1% vs. 12.9%, *p* = 0.04), and to be former smokers (34.4% vs. 16.9%, *p* = 0.04). The prevalence of HIV co-infection was lower in participants with TB and diabetes comorbidity compared with those with TB alone (18.8% vs. 41.8%, *p* = 0.001).

No significant differences were observed in the TB symptoms, history of TB treatment, type of TB treated, resistance to rifampicin, the pattern of chest X-ray findings, and anthropometric measures between both groups.

### Factors associated with tuberculosis and diabetes mellitus comorbidity

Table 2 summarises the factors associated with TB and DM comorbidity in this study population at logistic regression.

**Table 2:** Predictors of tuberculosis and diabetes mellitus comorbidity in participants with newly diagnosed tuberculosis

Covariates	Adjusted odds ratio	95% CI	P-value
**Age**			
< 40 years	1		
≥ 40 years	3.12	1.35–7.23	0.008
**Sex**			
Male	1		
Female	0.96	0.28–3.32	0.95
**Occupation**			
Unemployed	1		
Employed	2.96	0.91–9.65	0.07
**History of smoking**			
Yes	1		
No	0.91	0.34–2.4	0.84
**History of alcohol intake**			
No	1		
Yes	2.43	0.89–6.62	0.08
**HIV comorbidity**			
No	1		
Yes	0.27	0.10–0.74	0.01
**BMI**			
Normal	1		
Underweight	0.80	0.33–1.96	0.63
Overweight	0.55	0.05–6.13	0.63
Obese	3.54	0.46–27.15	0.22

Age ≥ 40 years was positively associated with TB and DM comorbidity (adjusted odds ratio or AOR 3.12, 95% CI 1.35–7.23, *p* = 0.008) while HIV co-infection was negatively associated (AOR 0.27, 95% CI 0.10–0.74, *p* = 0.01).

## Discussion

To our knowledge, this is the first study to investigate the burden of DM in adult patients with recently diagnosed TB using five screening and diagnostic tests (including an OGTT, the recommended gold standard test for diagnosing DM) in Uganda. In this study, most participants were diagnosed with DM using FBG measurement followed by laboratory-based HbA1c, OGTT, POC HbA1c, and RBG.

Compared with previous cross-sectional studies conducted in Uganda to determine the burden of DM in adult patients with TB, our study has reported the highest prevalence of DM in patients with TB. This could be because we used five screening blood tests, making the diagnosis of DM more robust.

Baluku JB et al. and Kibirige D et al. previously reported a high prevalence of DM in patients with TB of 6.5% and 8.5%, respectively, using RBG measurements [22, 27]. This discrepancy in the study finding could be explained by the differences in patient populations studied (patients with drug-resistant TB and hospitalised patients with TB in the former and latter studies, respectively). This is because of the differences in the TB severity (and related pro-inflammatory status) which influences the degree of the resultant dysglycaemia.

A comparable high prevalence of DM in adult African patients with TB has been reported in similar studies that have screened for DM using more than one test [14, 32–34]. Two case-control studies that screened for DM using FBG, HbA1c, and OGTT in patients with bacteriologically confirmed TB in South Africa [14] and Tanzania [33] reported an overall prevalence of DM of 12.6% and 16.7%, respectively. Contrary to what we observed in our study where the greatest proportion of patients was diagnosed based on an abnormal FBG level, HbA1c diagnosed more patients with TB and DM compared with FBG in one study conducted in Tanzania (9.3% vs. 4.5%) [32] and South Africa (10.2% vs. 4.4%) [14]. Despite using both RBG and FBG measurements as screening and diagnostic tests for DM in Ugandan adult patients with TB, Nsonga et al. reported a low prevalence of DM of 2.3% [26]. This lower diagnostic yield of HbA1c in Ugandan patients with TB in our study compared with the above-discussed studies may be related to specific patient factors that directly or indirectly affect the diagnostic performance of HbA1c like the presence of iron or vitamin B12 deficiency and haemoglobinopathies [35].

In our study, TB and DM comorbidity were associated with being ≥ 40 years old. Several studies have consistently reported that increasing age is associated with increased odds of having TB and DM comorbidity [5, 11–13, 16, 19, 36–39]. This may reflect reduced cellular and humoral immunity as age advances which increases the risk of developing both conditions [40].

Participants with HIV co-infection were less likely to have DM in our study population. The described effect of HIV co-infection on the association between TB and DM varies considerably in different African populations [14, 17, 19, 27, 33, 34, 41]. One cross-sectional study conducted on adult patients admitted with TB in Uganda [27] and two hospital-based studies conducted in Tanzania [19, 33] reported a reduced risk of DM in HIV-uninfected patients while two studies conducted in South Africa [14, 34] and one in Ethiopia [17] reported an increased risk of DM in HIV co-infected TB patients on stratification by HIV serostatus. In a systematic review and meta-analysis of 16 observational studies that investigated the prevalence of DM in TB patients in sub-Saharan Africa, a slightly higher pooled prevalence of DM was reported in HIV-infected participants compared with HIV-uninfected participants (8.9 [6.5–11.3]% vs. 7.7 [5.4–10.1]%, I^2^ = 78.3%, *p* < 0.001) [6].

The reasons to explain the protective effect of HIV against developing DM in patients with TB as demonstrated in our study findings and other studies conducted in Tanzania are unknown and need to be comprehensively explored. The increased risk of DM in patients with TB and HIV coinfection as reported in other studies may be related to the severity of HIV infection (advanced HIV disease with low CD4 count) which directly influences the pro-inflammatory state, insulin resistance, and occurrence of coinfections associated with pancreatic beta-cell dysfunction like hepatitis C infection. In addition, improvement in life expectancy due to the increased roll-out of antiretroviral therapy and their adverse effect of inducing metabolic disorders like dysglycaemia may explain the increased risk of developing DM in patients with TB and HIV coinfection [42–45].

In addition to what we observed in our study as predictors of DM in adult Ugandans with newly diagnosed TB, similar cross-sectional studies investigating the prevalence of DM in adult patients with TB have also reported sex [5, 11, 38], marital status [15], increased adiposity (based on body mass index or waist: hip ratio) [12, 15, 18], rural residence [13, 17], positive family history of DM [11, 17, 19, 38], and having pulmonary TB disease [38] as determinants of TB and DM comorbidity.

### Strengths and limitations

A major strength of this study was screening for DM using five blood tests including an OGTT which is the recommended gold standard test for diagnosing DM.

Despite this strength, the study had some limitations. Capillary blood instead of the recommended venous blood was used for the measurement of RBG and FBG. This may have introduced testing bias because, compared to venous blood glucose levels, capillary blood glucose levels are affected by numerous factors such as heat, temperature, and hypoxia and also give a higher blood glucose concentration. However, a new model glucometer that gives plasma-equivalent or calibrated capillary blood glucose levels was used to measure RBG and FBG levels in the study.

We only assessed the glycaemia at baseline without a follow-up component. The documented hyperglycaemia may have been transient with a possibility of reverting to normal glucose status at the end of TB treatment. This may be associated with a likelihood of over-reporting the prevalence of DM, especially in patients with transient hyperglycaemia that usually resolves at the end of treatment.

We did not evaluate for the presence of clinical and/or laboratory-determined anaemia using a full blood count in our study participants to detect its potential effect of affecting the performance of the HbA1c test.

We had a high proportion of participants (*n* = 42, 35.9%) not returning for re-testing. This could have led to the under-reporting of the burden of DM in this study population.

## Conclusion

Using the step-wise approach for diagnosing DM in patients with TB as recommended by the Union guidelines, TB and DM comorbidity were relatively common in our study population, especially in patients aged ≥ 40 years and HIV uninfected. Screening for DM should be encouraged in TB treatment centres to reduce cases of missed diagnosis.

### Electronic supplementary material

Below is the link to the electronic supplementary material.

Supplementary Material 1

Supplementary Material 2

Supplementary Figure 1: Glycaemic profile of participants with newly diagnosed tuberculosis

Supplementary Table 1. Participants diagnosed with diabetes mellitus based on each blood glucose test

## Data Availability

The datasets used and/or analysed during the current study are available from the corresponding author upon reasonable request.

## Abbreviations

AOR: Adjusted odds ratio
BMI: Body mass index
CI: Confidence interval
DM: Diabetes mellitus
FBG: Fasting blood glucose
HbA1c: Glycated haemoglobin
HC: Hip circumference
HOMA2: %B-HOMA2 beta cell function
HOMA2: IR-HOMA2 insulin resistance
IQR: Interquartile range
OGTT: Oral glucose tolerance test
POC: Point of care
RBG: Random blood glucose
TADIC: Tuberculosis and diabetes mellitus comorbidity
TB: Tuberculosis
WC: waist circumference
WHR: waist: hip circumference ratio
