# Supplementary material for: Tuberculosis and diabetes mellitus comorbidity in an adult Ugandan population
Source: BMC Infect Dis. 2024 Feb 22;24:242. doi: 10.1186/s12879-024-09111-8 (PMC10885501; doi:10.1186/s12879-024-09111-8)
Supplement: Supplementary file 1 — Supplementary Material 1 [file 12879_2024_9111_MOESM1_ESM.docx]

**Supplementary Figure 1. Glycaemic profile of participants with newly diagnosed tuberculosis**

232 participants initially screened using random blood glucose (RBG) testing

DM diagnosed in **3 participants (4.1%)** with a point-of-care HbA1c level ≥6.5%

- Diabetes mellitus (DM) diagnosed in 32 participants (13.8%)
- Pre-diabetes diagnosed in 65 participants (28.0%)
- Normal glucose status confirmed in 135 participants (58.2%)

DM diagnosed in **7 participants (9.5%)** with a 2-hour blood glucose level ≥11.1 mmol/l after OGTT

DM diagnosed in **22 participants (29.3%)** with a FBG level ≥7 mmol/l

DM diagnosed in **8 participants (10.7%)** with a laboratory-based HbA1c ≥6.5%

75 participants (64.1%) return for measurement of the fasting blood glucose (FBG), glycated haemoglobin (HbA1c), and oral glucose tolerance test (OGTT).

115 participants (49.6%) with RBG level <6.1 mmol/l (**not** **requiring further blood glucose tests**)

117 participants (50.4%) with RBG ≥6.1 mmol/l (**rescheduled for re-testing of the blood glucose levels**)
