# Supplementary material for: Tuberculosis and diabetes mellitus comorbidity in an adult Ugandan population
Source: BMC Infect Dis. 2024 Feb 22;24:242. doi: 10.1186/s12879-024-09111-8 (PMC10885501; doi:10.1186/s12879-024-09111-8)
Supplement: Supplementary file 2 — Supplementary Material 2 [file 12879_2024_9111_MOESM2_ESM.docx]

**Supplementary Table 1. Participants diagnosed with diabetes mellitus based on each blood glucose test**

| **Blood glucose test used** | **Participants diagnosed with diabetes mellitus (n)** | **Prevalence of diabetes mellitus (%)** |
| --- | --- | --- |
| Random blood glucose (n=232) | 5 | 2.2 |
| Point-of-care glycated haemoglobin (HbA1c) (n=73) | 3 | 4.1 |
| Oral glucose tolerance test (OGTT) (n=74) | 7 | 9.5 |
| Laboratory-based glycated haemoglobin (n=75) | 8 | 10.7 |
| Fasting blood glucose (FBG) (n=75) | 22 | 29.3 |
| FBG and OGTT (n=74) | 4 | 5.4 |
| HbA1c and OGTT (n=74) | 1 | 1.4 |
